# Transtubular Transoral Surgery for Excision of a Dystrophic Os Odontoideum: A Case Report

**DOI:** 10.5704/MOJ.1603.009

**Published:** 2016-03

**Authors:** MH Ariffin, MM Ashfaq, E Kang

**Affiliations:** Department of Orthopaedics and Traumatology, Universiti Kebangsaan Malaysia Medical Centre, Kuala Lumpur, Malaysia

**Keywords:** Transtubular, Transoral, Os Odontoideum

## Abstract

Transoral approach to the cervico-medullary junction is a well-established procedure. However oropharyngeal complications in the form of soft tissue morbidity postoperatively do occur. We report a case of a teenage boy with traumatic quadriparesis secondary to compression of the cervico-medullary junction by an os odontoideum. Decompression was done via transoral approach through a tubular retractor system, hence obviating the need for the splitting or separate retraction of the soft palate and minimised the damage and violation of surrounding soft tissues. His neurological status improved and he was able to ambulate with support on fourth post-operative day with no soft tissue morbidity in the oral cavity. To our knowledge this is the first case reported using this technique. We conclude that adoption of this method would improve the traditional transoral approach and reduce the oropharyngeal complications.

## Introduction

Transoral approach was first described by Fang and Ong in 1962. The high complications rate associated with this approach tempered many surgeons to shy away from it. With the advancement and introduction of better surgical tools and imaging modalities, this approach is now a well-established procedure. Despite the advancements, the procedure still carries significant risk of damage to surrounding structures causing complications such as dysphagia, speech difficulties, laryngeal swelling or hematoma, vessel injury or neurological deficit.

## Case Report

A 14 year-old boy was seen in our centre after trauma sustained during martial art training school. He was thrown down and landed on the top of his head. Immediately, he complained of midline neck pain with bilateral upper and lower limbs numbness and weakness causing him inability to stand and walk. There was no urinary and bowel incontinence.

**Fig. 1:**
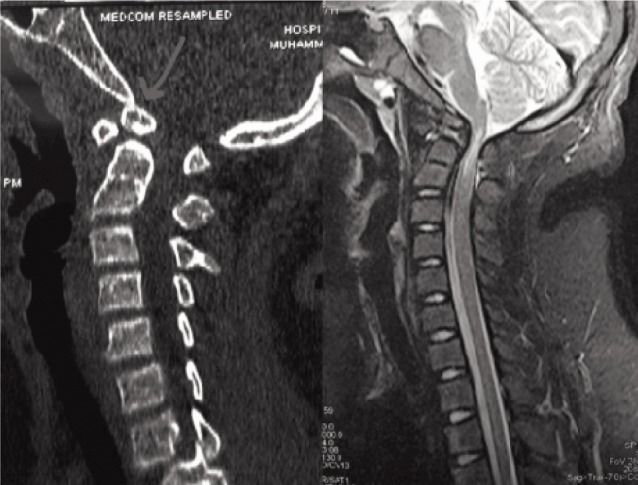
CT scan showing dystrophic form of os odontoideum. MRI showing the cervico-medullary cord compromise.

On admission, the patient was conscious with rigid cervical collar in place. Vital signs were within normal range. The patient had palpable midline tenderness over the upper cervical region. Neurologically his left upper limb was weak with motor power of C5 : 4/5, C6 : 3/5, C7 : 2/5, C8 : 3/5, and T1 : 1/5. The right upper limb power and both the lower limb power were grade 3/5. The computed tomography (CT) scan revealed an os odontoideum. MRI showed a marked angulation at the cervico-medullary junction, with more than 50 percent compromise of the available space for the spinal cord, and associated cord oedema (Figure 1).

**Fig. 2:**
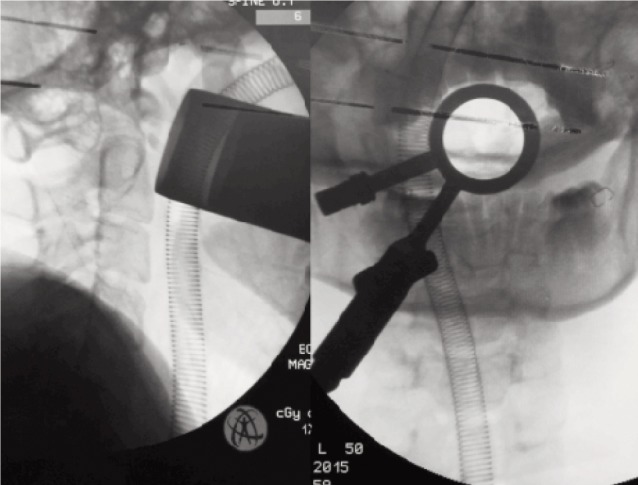
Placement of the tubular retractor through the oral cavity and its confirmation on the intra operative C-arm image.

**Fig. 3:**
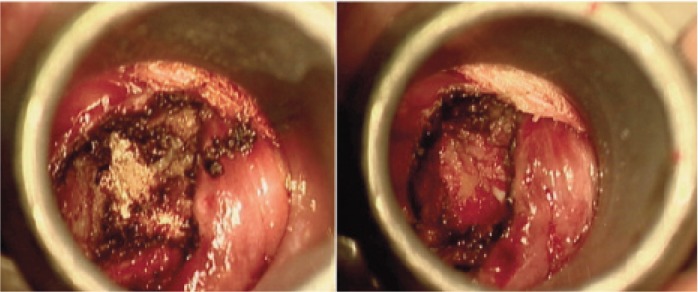
Intraoperative exposure of the anterior arch of C1 through tubular retractor system and the completed decompression after removal of the dystropic os odontoideum.

The patient underwent transtubular transoral resection of the os odontoideum followed by posterior C1-C2 wiring. He was operated in a supine position with 30 degree head-up inclination. A 90-mm tubular retractor was inserted through the oral cavity and directed directly onto the C1 anterior arch and the position was confirmed with a lateral view and AP intraoperative image intensifier (Figure 2). The tube pushed superiorly the uvula and the soft palate as it docked down on the posterior pharyngeal wall. It was then secured to the side rail of the table. An operating microscope provides illumination and allowed excellent visualisation through the tube. A midline incision was made over the posterior pharyngeal wall, the layers separated and the anterior tubercle of the C1 exposed (Figure 3). To access the os odontoideum the middle 1.5 cm of the anterior arch of C1 was removed with a high speed burr. The os was then removed and the spinal cord decompression was completed (Figure 3). It was complicated with a dural leak dura which was secured with Tissel. The blood loss was only 50ml. The posterior pharyngeal wall was sutured with three interrupted sutures. The patient was then placed in prone position for C1C2 wiring and bone grafting. The surgery lasted 160 minutes. The patient was sent to the ICU and extubated the next day. He was started on liquid diet on the second postoperative day and soft diet was started on the fifth post op day. The oral hygiene was maintained with two-hourly Chlorhexidine gargles.

The patient regained full power and sensation of the right lower limb but the left lower limb improved to grade 3-4/5. Power of both upper limb improved to 3-4/5 but the left T1 remained at 1/5. Post operative CT scan confirmed compete excision of the os odontoideum (Figure 4). He was able to ambulate with forearm support and to transfer himself from the bed to the wheelchair by the fourth day post-operation. During follow up at three months post operatively, he had full neurological recovery.

## Discussions

Os odontoideum is an independent ossicle of variable size with smooth circumferential margins separated from a fore-shortened odontoid peg. The ossicle stands apart from the hypoplastic dens and can adopt two anatomic types: orthotopic and dystopic.^1^ An ossicle located in the position of the normal odontoid is referred to as orthotopic. The ossicle is considered dystopic if it appears near the occiput in the area of the foramen magnum. The incidence of os odontoideum is unknown because the lesion is usually asymptomatic.^2^ The presentation of os odontoideum can vary from an incidental radiographic finding to severe findings of myelopathy, vertebral artery compression, and intracranial manifestations. The primary concern in this odontoid abnormality is the ability of the abnormal atlantoaxial joint to subluxation or even dislocate with minor trauma, resulting in death or permanent neurological damage.

Our patient experienced quadriparesis from cervicomedullary compression following trauma. This necessitated transoral decompression and resection of the os odontoideum followed by posterior fixation to stabilize the upper cervical spine. The conventional transoral approach requires inversion of the soft palate into the nasopharynx or soft palate splitting which has post-operative oropharyngeal morbidity. There are many differing views on the techniques to achieve superior intra-operative exposure and avoid injury of surrounding structures in transoral anterior approach, or preoperative tracheostomy versus oral or nasal intubation and incision of soft palate or retraction.^3^ The latter procedure predisposes patient to rhinolalia, nasal regurgitation and palatal wound dehiscence.^4^

However, the use of the tubular retractor is simple and fast as the approach is made merely by inserting the right length tubular retractor direct into the mouth. The tube can then be further angulated to facilitate access by what is popularly known as “wanding” in low back MIS surgery. It is minimally invasive as there is no need to split the soft palate as the tubular retractor naturally pushes away the soft palate, the uvula and the pillars of the tonsils as it “docks” down on the posterior oropharynx. All the complications associated with the traditional splitting of the uvula or incising the soft palate is avoided while maintaining the view of the lower clivus and the craniovertebral junction for the spinal decompression in our case. The tube also protects the tongue, soft and hard palate, tonsillar areas that can be injured during traditional transoral as the tube acts as a working portal. The use of a surgical microscope not only provide excellent illumination and magnification but also provides better appreciation of depth and 3D visualisation compared to an endoscope.

Transoral surgery does not fully destabilise the spine; however this operations may potentiate incipient pathological instability. The primary determinants of instability are the extent of the pathological bone destruction, ligamentous weakening and operative bone removal. Therefore one-stage combined transoral decompression and posterior fixation is recommended.^5^

In summary, os odontoideum is an uncommon, but important cranio-cervical spine abnormality that can be associated with significant spinal cord stenosis. Traditional transoral route has proven to be an invaluable method of approaching pathologies at cervico-medullary junction but the oropharyngeal complications are greatly outweighed by the benefits of the surgery. Such dreaded injuries could be easily avoided by the use of a tubular retractor.
